# How gender shapes interprofessional teamwork in the operating room: a qualitative secondary analysis

**DOI:** 10.1186/s12913-021-07403-2

**Published:** 2021-12-19

**Authors:** Cole Etherington, Simon Kitto, Joseph K. Burns, Tracey L. Adams, Arija Birze, Meghan Britton, Sukhbir Singh, Sylvain Boet

**Affiliations:** 1 Clinical Epidemiology Program, Ottawa Hospital Research Institute, 501 Smyth Rd, Ottawa, ON K1H 8L6 Canada; 2 Department of Anesthesiology and Pain Medicine, The Ottawa Hospital, University of Ottawa, General Campus, 501 Smyth Rd, Critical Care Wing 1401, Ottawa, Ontario K1H 8L6 Canada; 3 Department of Innovation in Medical Education, University of Ottawa, 451 Smyth Rd, Ottawa, ON K1H 8M5 Canada; 4 Department of Sociology, Social Science Centre, Western University, London, ON N6A 5C2 Canada; 5 Dalla Lana School of Public Health, University of Toronto, 155 College St, Toronto, ON M5T 3M7 Canada; 6 Main Operating Room, The Ottawa Hospital, 501 Smyth Rd, Ottawa, ON K1H 8L6 Canada; 7 Department of Obstetrics, Gynecology & Newborn Care, University of Ottawa, 501 Smyth Rd, Ottawa, ON K1H 8L6 Canada; 8 Francophone Affairs, Faculty of Medicine, University of Ottawa, 451 Smyth Rd, Ottawa, ON K1H 8M5 Canada; 9 Faculty of Education, University of Ottawa, 145 Jean-Jacques-Lussier Private, Ottawa, ON K1N 6N5 Canada

**Keywords:** Gender role, Health personnel, Qualitative research, Professional practice, Operating rooms, Social environment

## Abstract

**Abstract:**

**Background:**

Despite substantial implications for healthcare provider practice and patient outcomes, gender has yet to be systematically explored with regard to interprofessional operating room (OR) teamwork. We aimed to explore and describe how gender and additional social identity factors shape experiences and perceptions of teamwork in the OR.

**Methods:**

This study was a qualitative secondary analysis of semi-structured interviews with OR team members conducted between November 2018 and July 2019. Participants were recruited across hospitals in Ontario, Canada. We conducted both purposive and snowball sampling until data saturation was reached. Transcripts were analyzed thematically by two independent research team members, moving from open to axial coding.

**Results:**

Sixty-six interviews of OR healthcare professionals were completed: anesthesia (n=17), nursing (n=19), perfusion (n=2), and surgery (n=26). Traditional gender roles, norms, and stereotypes were perceived and experienced by both women and men, but with different consequences. Both women and men participants described challenges that women face in the OR, such as being perceived negatively for displaying leadership behaviours. Participants also reported that interactions and behaviours vary depending on the team gender composition, and that other social identities, such as age and race, often interact with gender. Nevertheless, participants indicated a belief that the influence of gender in the OR may be modified.

**Conclusions:**

The highly gendered reality of the OR creates an environment conducive to breakdowns in communuication and patient safety risks in addition to diminishing team morale, psychological safety, and provider well-being. Consequently, until teamwork interventions adequately account for gender, they are unlikely to be optimally effective or sustainable.

## Introduction

Suboptimal teamwork in the operating room (OR) increases the risk of surgical errors and complications for patients by up to 14 percent [1]. Effective teamwork can be threatened by a variety of multi-level factors, including power and hierarchy [2–4]. Hierarchical relationships have often been investigated in the OR setting, typically with regard to trainee or professional status, such as interactions between staff physicians and residents or nurses [5, 6]. In reality, hierarchical relationships can be influenced by a variety of sociocultural norms related to multiple axes of social difference, including gender [7]. As opposed to sex, which refers to biological attributes, gender is a multifaceted and fluid construct [8]. Gender refers to the expressions and identities of women, men, and gender diverse people as well as the socially constructed norms and roles applied to individuals based on their ascribed or experienced gender [8]. This also includes how individuals perceive, evaluate, and interact with each other [8].

Recent literature in surgery and anesthesia has drawn attention to the importance of gender in shaping the experiences and outcomes of healthcare professionals [9–16]. For example, although the number of women entering medical school has surpassed that of men, women still remain under-represented in many specialties [17]. Studies have also documented women physicians experience bias and harassment [17, 18], particularly in surgery and anesthesia, and as a result, may be more susceptible to burnout as a result [19]. At the same time, evidence suggests the individual practice patterns of men and women physicians may vary considerably, and as a result, patient outcomes may also vary [20–25].

Although gender has been increasingly explored at the individual healthcare provider level, its role within the context of the interprofessional OR team is complex and requires further exploration for deeper understanding. This may have important implications for interprofessional education and training, particularly with regard to the growth and increased investment in equity, diversity, and inclusion [26]. Given that ineffective teamwork continues to be a primary contributing factor to surgical patient complications [3], there is a need to consider how traditionally overlooked factors, such as gender, affect interprofessional practice in the OR. This qualitative study therefore aimed to explore and describe how gender and related social factors shape teamwork from the perspective of the different OR professions.

## Methods

This study is reported according to the Consolidated Criteria for Reporting Qualitative Research (COREQ) checklist [27].

### Study design

This study is a qualitative secondary analysis of primary interview data collected for an initial related project. This study was approved by the Ottawa Health Science Network Research Ethics Board on February 8, 2018 (#20170875; coordinating site) and the Unity Health Toronto Research Ethics Board (#18-396) on February 12, 2019.

#### The primary study

Data were originally collected during our initial qualitative interview study which aimed to determine barriers and enablers to effective teamwork [28]. The semi-structured interview guide was based on the Theoretical Domains Framework (TDF) [29, 30]. Briefly, the TDF is a comprehensive framework derived from 33 theories of behaviour and behaviour change, synthesized into 14 domains, which has frequently been used in implementarion research to explore barriers and enablers to desired behaviours [29, 30]. The interview guide was piloted with one surgeon and one Registered Nurse (RN) to ensure questions were clear and interpreted consistently across groups. Interviews were conducted with interprofessional OR team members between November 2018 and July 2019. All interviews were conducted by the two interviewers (CE, JKB) in English and lasted approximately one hour. Each interview was audio-recorded and transcribed. All recordings were de-identified and deleted following transcription.

We used purposive sampling [31] at each of the participating sites, which involved recruitment emails administered through the various perioperative departments of four academic hospitals located in Ottawa, Canada and Toronto, Canada. Each of these sites performs a large volume and variety of surgical procedures, allowing us to account for potential variations by OR culture. As teaching hospitals, these sites also allowed us to recruit surgical and anesthesia post-graduate trainees. Obtaining a diverse sample when conducting qualitative research is important for enhancing its quality and trustworthiness, particularly when exploring complex phenomena [32].

We also engaged in snowball sampling [31], where participants were invited to refer their colleagues to the study. We determined *a priori* that we would conduct a minimum of 10 interviews of women and 10 interviews of men within each major professional group (anesthesia, nursing, surgery) to explore variability within groups and identify important nuances. The final sample size was then determined based on thematic saturation [33]. When three interviews [33] were conducted without the emergence of any new themes within each gender and professional group, recruitment ended. Due to challenges in recruiting women surgeons, women and men anesthesiologists, and men nurses, we were unable to apply our *a priori* criteria of “10+3” [33] interviews; however, themes were consistent across interviews, with no new themes emerging after analysis of the first three interviews for men nurses, and the first six interviews for each of the physician groups.

#### The current study: qualitative secondary analysis

For the purposes of the present study, data were re-analyzed to specifically explore themes related to gender and additional social categories that emerged as relevant according to participants (e.g. race, age). Secondary analysis of qualitative data allows researchers to “maximize data utility”, especially with populations such as busy healthcare professionals, where it can be difficult to recruit large samples for multiple interview studies [34]. In addition, because qualitative research requires a significant investment of resources, secondary analysis provides an efficient and cost-effective way to apply a new perspective and expand findings from the original data, rather than having to collect data from new subjects [34]. Although there are times when secondary analysis of qualitative data may not be appropriate (e.g. unrelated research question, extended period of time between original and secondary analysis), it was well-suited for the current study for several reasons. During data collection and analysis for the original study, it became apparent that gender was an important factor in shaping teamwork experiences in the OR. This helped to formulate the research questions for the secondary analysis reported in this manuscript, rendering the secondary topic closely aligned to the first as these questions represent an important aspect of the primary phenomenon (OR teamwork) that was originally studied. Our secondary analysis extended the original study to recognize and further explore the role of gender and additional social factors in the OR, using an established relevant cohort of participants. This exploration can be leveraged in the future to support further in-depth study of gender and OR teamwork. It should also be noted that the secondary analysis took place immediately after the original analysis and was conducted by the same core research team members.

### Researcher characteristics and reflexivity

The co-investigator team collaboratively developed the study protocol, drawing upon their interdisciplinary expertise in sociology, gender studies, psychology, medical education, and implementation science as well as clinical experience (surgery, anesthesia, nursing). The diverse expertise amongst the team helped to both enrich data analysis and interpretation and to ensure evaluative rigour [35]. The team critically considered how their various intersectional identities (e.g., belonging to various BIPOC, official language minority, 2SLGBTQ+, geographic, and professional communities) may have shaped the research process through reflexive discussion at each stage. This helped to ensure a comprehensive and inclusive study protocol and interview guide in addition to trustworthiness of the results [36]. For example, the team discussed how their own interprofessional OR experiences related to gender and intersecting social identities, and found that their professional experiences largely matched the themes arising from participant responses, enhancing confirmability of the results [36].

Interviews and data analysis were conducted by two research team members (CE, JKB), who also self-identified at various intersecting social locations. These team members were “outsiders” in that they were not clinicians and had no preconceived notions of what takes place everyday in the OR. They had no previous relationships to the participants and were trained in qualitative interview techniques. During interview coding, the team members regularly discussed how their diverse educational backgrounds (sociology and biomedical science) and social locations brought different yet complementary perspectives to the analysis, ensuring data were considered from various angles.

### Data management and analysis

De-identified transcripts were imported into NVivo Version 12 (QSR International, Doncaster, Australia), a qualitative analysis software.

Transcripts were analyzed thematically [37] by two independent research team members (CE, JKB). Open coding was first conducted with three interview transcripts to identify initial concepts related to the research question. Coding was then reviewed by the two coders together and discrepancies were resolved through discussion. A coding strategy was subsequently developed. After establishing reliability (weighted Cohen’s kappa > 0.80), the remaining transcripts were divided equally between the two coders. To ensure reliability was maintained throughout the duration of the analysis period, 10% of interviews were coded in duplicate and discussed regularly to maintain interpretive rigour [35].

Following the open-coding process, one research team member (CE) conducted axial coding [38], which involved clustering of similar codes (i.e. units of meaning). Codes were then further developed and refined to identify patterns of meaning within and across interview transcripts, both by professional group and as a whole (i.e., across all three professions). Transcripts were analyzed one subject at time regardless of profession, and then themes were revisited to look for profession-specific nuances. Themes identified through this process were then reviewed by a second research team member (JKB), which led to further refinement and collapsing of core themes. Findings were then reviewed by each member of the interdisciplinary research team to develop the final structure of key themes and relationships between them.

### Trustworthiness

Steps were taken at each stage of the research project to meet established criteria for trustworthiness, namely, credibility, dependability, confirmability, and transferability [39]. To achieve credibility, our team engaged with participating sites for several months during the original study and pilot tested the interview guide. We also ensured members of the research team were knowledgeable in OR teamwork, gender and intersectionality, and qualitative research. To achieve dependability, we prepared a detailed study protocol and assessed inter-coder reliability. To achieve confirmability, we engaged in reflexivity and investigator triangulation. To achieve transferability, we used a combination of sampling techniques and quantified data saturation.

## Results

### Participant characteristics

Participant demographics are summarized in Table 1. Of the 66 participants, 36 (55%) were women, while the remainder (n=30[45%]) identified as men. There were no participants who identified as transgender or nonbinary. Of the 17 anesthesia participants, eight were staff, seven were residents, and two were fellows. There were 13 staff surgeons, 11 surgical residents and two surgical fellows who participated, along with 23 nursing staff members. The median participant age was 35 (interquartile range=29-43). Most of the participating attending and post-graduate trainee surgeons (n=26) specialized in general surgery (n=13; 50%). Three surgeons (11.5%) specialized in urology and three specialized in orthopedics. One surgeon (3.8%) specialized in each of the following specialties: gynecology/obstetrics, neurosurgery, otolaryngology, oncology, plastics, and trauma. The majority of participants identified as Caucasion (n=51), and the median participant age was 35 years (Table 2).

**Table 1:** Profession and gender identity of participants

Profession	n (%) women	n (%) men	n (%) transgender or nonbinary	Total
Anesthesia	9 (53%)	8 (47%)	0	17
Nursing*	15 (79%)	4 (21%)	0	19
Perfusion	1 (50%)	1 (50%)	0	2
Surgery	10 (38%)	16 (62%)	0	26
**Total**	36 (55%)	30 (45%)	0	66

*Includes Registered Nurses (RNs) and Registered Practical Nurses (RPNs), and Perfusionists.^1^

^1^In Canada, RPNs receive a 2-year college diploma and are licensed to treat stable, predictable or non-severe conditions while RNs receive a 4-year Bachelor’s degree and can attend to more complex health issues. In the OR, RPNs are able to practice as Scrub Technicians while RNs may practice as the circulating nurse.

**Table 2:** Age and ethnicity of participants

Age group (years)	n (%)	Ethnicity^a^	n (%)
20-25	3 (5%)	Asian	9 (14%)
26-30	19 (29%)	Black	2 (3%)
31-35	13 (20%)	Caucasian	51 (78%)
36-40	9 (14%)	Hispanic	1 (1%)
41-45	9 (14%)	Middle Eastern	2 (3%)
46-50	6 (9%)	Not reported	1 (1%)
51-55	4 (6%)		
56-60	1 (1%)		
>60	1 (1%)		
Not reported	1 (1%)		

^a^as described by participants

### Interview themes

Themes and sub-themes identified from the interview data, along with illustrative quotes, are reported in Table 3 and summarized below. We identified four main themes: “women face challenges in the OR – and everyone experiences or observes it”; “gender impacts team interactions or behaviours”; “several social identities intersect with gender to shape teamwork interactions”; and “the influence of gender in the OR can be modified”.

**Table 3:** Identified themes and sub-themes from semi-structured interviews with OR team members

Theme and sub-theme	Illustrative quotes
**1. Women face challenges in the OR – and everyone experiences or observe it**	
***Experienced across professions***	
Harassment and bullying	We have what we call a blue form, so you can blue form any employer, any staff, which means, it's like a warning. It's like any type of harassment or violence, or aggression, anything and it's done anonymously. There are a couple of handful of male surgeons that have been blue formed by their colleagues a number of times and they still keep doing whatever they’re doing. It’s like, oh it’s okay because it’s him. It’s so and so. That’s how he is. Well, that’s not an excuse for him to keep doing that. Right? - Woman RN 012
Having to accept “how it is”	I guess I’ve learned a lot about how to interact with male surgeons over the years... I remember when I started practice that it was definitely, you know, something you think about. I thought about it a lot and sort of how to... I learned a lot about that when I was younger… You know, just being pushed around. Yeah. Like, you know, you want to do this case and you want to do that, and what do you mean, you want to cancel, and why do you want to cancel. And I always sort of felt like defensive. And I don’t anymore at all. So, I just say, “This is how it is” … - Woman anesthesiologist 009
Proving yourself	You have to work so much harder to prove yourself, that you’re legit. You’re good at what you do and have people respect you. So, yeah, being a female and also being a nurse, nurses are always kind of underlooked. I think it makes it harder on us to communicate, to be respected, to be heard by everybody. – Woman RN 008
	I think that, as a male surgeon, I probably got a bye early on. I think my female colleagues probably have more of an uphill battle in the early days to establish themselves as the, you know, surgeon in the room, the leader in the room. So, I think we start out… I think at the beginning of the career, the women are at a disadvantage… I think all comers first day in the OR, a man probably has an easier time. – Man surgeon 016
Women taken less seriously/not listened to	…I could be working on a case for three hours, telling the surgeon… “I’m concerned about the bowel.” And the surgeon’s, like, “Okay, okay, okay, okay.” And another Fellow... so exactly of my training, male, tall, would come in and, you know, chit-chat a little bit… And then the Fellow... would say, “Whoa, look at that valve! Look at the gradient!” And the surgeon’s, like, “What? Oh, I didn’t realize it was that bad.” And then, honestly? Change the entire surgical plan and go into fix that valve. And I found [it] infuriating because I was telling them that. And there were examples in Fellowship where... I can only say it was gender because other Fellows of my exact same training who were male would come in and say the exact same finding, and the surgeons would listen… And meanwhile, you know, I’m using all of my possible red flags of concern and they’re not listening… [and even] if I was with a female attending, honestly, we would have to call in a male attending sometimes to give the exact same finding for the surgeon to look into it, to change the surgical plan. – Woman anesthesiologist 003
	…so I was loading up a new scalpel blade for [the surgeon] because he wanted a fresh one. While I was doing that, he asked for three other instruments and I was like, “Okay well give me a sec, let me load this up first, I can get that for you in just a sec.” …and he couldn't wait for a second. So, I look up for a quick second because I see three arms going across my surgical table and most people know you're not supposed to touch the scrub nurse's table. That's off-limits, you need to ask for permission. I just see three hands going over, the resident's and the fellow… and the next thing I knew I had cut myself with the blade because I didn't load it up properly because I was rushing to tend to them and to see what they were grabbing or whatever. So, now I have a deep laceration, I have seven stitches because of it. – Woman RN 002
Differential respect	Yeah…it still comes across as very kind of patriarchal sometimes when you look at how... I mean, especially the older physicians, 'cause that’s kind of the environment they were raised in, but... whether it’s speaking to the female nurses or other female physicians or whatever it is, sometimes they kind of come across as very... patriarchal as opposed to when a man is talking to a man, or when they talk to me, it’s this weird kind of like... like mutual respect, I guess? As like, “Oh, well, you’re... you’re also a man and so I can talk to you normally.” – Man RN 001
Lower confidence in decisions as a trainee	A lot of the time I have some clinical decisions that I have made in my mind, but I have some doubts regarding these decisions, and so I wait until I've discussed them with my staff before implementing the decision… so I have been told by many female staff that this hesitation is likely coming from being less confident… and that they felt the same way for many years before really deciding that they were going to speak up or they were going to do that or so on… as opposed to many, many male trainees who make a decision and right away act on it with a lot of confidence. – Woman anesthesiologist 005
“Boys club” mentality	[teamwork] It’s a... it’s a gender thing. It’s sort of, you know, it’s... it’s that, still, it’s a, you know, the boys club or something. Especially with the older male surgeons and, you know, and, or males in the OR sort of thing. – Man RN 003
Women are often outnumbered	I can’t think of any other contributor to why they wouldn’t listen to me and they would listen to the other, male Fellows. Working in an environment that’s, like, hugely male-dominant, there’s no female cardiac surgeon there. There’s [very few] female anesthesiologists [compared] to male anesthesiologists. – Woman anesthesiologist 001
***Specific to nursing***	
Fear of speaking up to male surgeons	I would say watching a lot of the newer nurses come in, a lot of them are scared, terrified to speak up, to speak up to the surgeon. And I would say in a sense I’m still working on that, because there is that tension and that ‘oh *he’s* the surgeon', and whatever he says is going to be right. This may be a silly question, I'm afraid to ask. You know, that hesitancy you have... Terrified to talk to the surgeons or ask anything. Now after seven years, I’m going to ask and just be upfront. Sometimes it’s still hard, it depends on who it is, right? But definitely, for lots of new nurses and most of them are female, it is super hard. – Woman RN 013
Tension with women surgeons	… I find some females are trying so hard to put themselves and make themselves… assertive... I think because it's such a male-dominated field… I think they have to, like, assert themselves in that field, like, in their role, but it comes across as, like, almost b*tchiness, I don’t know if that’s the right term… and it’s like whoa, I’m a female too... like I get we’re working with all males, they don’t need to... they don’t need to be rude to me, I didn’t do anything. – Woman RN 015
***Specific to surgery***	
Impression management; performativity	We say that, you know, as a female surgeon, you're either a pushover or you're a b*tch. Like you kind of have to be one or the other, that you can't just be neutral because you just sort of get… this sort of attitude of people are less willing to do things for you than your male colleagues… And I think being on staff you… there's a power dynamic that you have regardless but really trying hard not to be-- not to be labelled as being b*tchy and being overly demanding. I would say there's a lot of things that I do for myself that I am very sure that none of my male colleagues do for themselves… like getting equipment, planning ahead and making sure like if I need something extra that I will go do it myself or go get it myself to make sure it's there. Or if the case happens and you ask for it and people don’t listen, you just kind of have to ask again nicely. There's no… yelling for things. – Woman surgeon 010
Cases not prioritized	…the Plastic Surgeon I was telling you about… when it comes to urgent bookings, there’s a policy to it, right? Like how urgent is this case? …So, like the surgeons will fight at the desk, they’ll say, “Oh no, my patient needs to go ahead of yours because this is more urgent or because this and this.” I see it, this Plastic Surgeon, she oftentimes gets bumped by people. I mean, there’s one thing being bumped by just the nature of how sick the patient is. That’s understandable. But there’s another reason bumped because, ‘It’s Dr. [name]…’ I see a lot of male surgeons that talk bad about her behind her back because she’s got a loud personality and she’s so particular. – Woman RN 014
Assertive women surgeons are perceived negatively by others	I think... I’ll just say that I think at baseline that... for, if the same words, the same actions, that are performed or said by me, they are-- that are done by a female surgeon, that female surgeon can be viewed... I would be more likely to be viewed as assertive, authoritative or a strong leader, but a female surgeon is very much likely to be viewed as, you know, hostile... I’m not going say the ‘B’ word, but... you know, that’s all real. – Man surgeon 004
Tension with women nurses	… I find the assumption, when it’s like a female-to-female interaction, is that you are bossy or aggressive or think you know it all or think you’re better than them. And so if you kind of take the stance of, “I’m just gonna bring myself down,” then people kind of don’t see you as a threat. But it’s sad that you have to do that… Like, I have to consciously represent myself as, like, “Please help me. I don’t know what I’m doing.” As opposed to, like, any sort of confidence and expertise is perceived as a threat. And I think that, especially in the OR environment... and I will say especially a female Surgery resident. Often there’s a lot of kind of pushback from the Nursing staff and it becomes sort of important to kind of form an ally rather than have an enemy. … I think that also the interaction female-to-female is a much more difficult interaction than male-to-female or male-to-male. …you know, the stereotype of a surgeon is an old white male. And the stereotype of a nurse is a young female. And I think when there’s anyone that is not in those roles, the dynamic can be different. So, having a young female surgery resident… it’s more difficult and you have to be more sort of conscious in your efforts to form it than some of our male colleagues have to. – Woman surgeon 009
***Specific to physicians (anesthesia or surgery)***	
Adapting specific communication strategies	And I would say I’ve had to adapt different ways of engaging team members than maybe some of my other colleagues and that’s based on advice I’ve had from more senior female anesthesiologists. So using language more carefully as opposed to posture and sort of just implying that you’re in control. So escalating your language to be like ‘I am concerned,’ I think that… being very explicit. Other things that I had that work is if I stand on a step stool so that I’m higher (laughs). And just sort of like verbal and nonverbal cues that I don’t think my male colleagues are using. – Woman anesthesiologist 007
Not being perceived as physicians	It’ll be myself and a male medical student and, you know, I’ll tell the patient all about their surgery and then they’ll turn to the male and say, like, “What’s your opinion, doctor?” – Woman surgeon 005
**2. Gender impacts team interactions or behaviours**	
Co-worker vs. friend	…when you have kind of two guy nurses working in the same room, it’s like working with your buddy as opposed to working with a co-worker more… yeah, it’s kind of a weird... when you’re a kid you have more guy friends than you do girlfriends and so when you enter a work environment and you’re working with another guy, it’s like, “Oh, yeah, you’re my…” like, you’re my buddy, you’re not my co-worker in the same... in the same sense as it would be with somebody of the opposite gender. – Man RN 004
Male nurse – male physician interaction	I’m a man nurse, which is, on its own, not as common, but we’re definitely a lower population. And so that... it kind of changes the dynamic being a male nurse in an environment where you’re different from everybody else who’s on your team, but then on the flip side, a majority of the surgeons and anesthesiologists are male, so I find, being a man in an environment where it’s primarily men, there’s kind of automatically that understanding, I find, or some innate communication style that men have with each other… it helps to communicate with the other teams, being a male, but then it also changes the dynamic... the dynamic because I’m a male who isn’t a doctor in that environment. And it’s kind of bizarre. ‘Cause it helps, but then it... it still puts me in kind of a weird position because a lot of the time they’re talking kind of man-to-man with other physicians and so, when I kind of enter that realm, it’s like I’m one of them but not one of them. – Man RN 001
Men communicate more directly	And so I don’t think it's an advantage, you know, it's getting better because there's more and more males working in the operating room here. One thing I do notice, there's less bickering when there's more males around. So there's more communication and it's more direct, to the point than p***ing around the pot. – Man RN 002
Men can be perceived by others as intimidating	So even though I’m not willingly, or hopefully not even unconsciously trying to behave in a way that influences teamwork, I might be perceived as intimidating. And my gender therefore would not help in that. If I’d be a petite woman with a soft voice, I’m sure I would not be as easily perceived as intimidating. So even though my intentions are not that way, I can see that being a man influences that. – Man anesthesiologist 004
Women can be perceived as less dominant	I always find it easier to interact with women in the operating theatre. I think that women are less likely to try and assume control or be domineering. – Man anesthesiologist 002
Women are soft-spoken; men are loud	Yeah, I think just, for me, personally, I am soft-spoken, but not all females are soft-spoken. There’s definitely some females that you can hear very loudly. But I think we, as a gender, females, are less loud than males. And that does affect teamwork. – Woman anesthesiologist 008
Different flow depending on who you are working with	Well I wouldn’t say, like, physically, because they’re females, they’re weak or something, but you know, but in terms of, like, when you’re working the Ortho, which is more, you know, like, there’s more demands about physical work, I’m not saying that some... some... I’m not saying that, you know, but you have, you know, older generations of coworkers that still works in Ortho and... so, but definitely the flow, the pacing, and when you work with someone, like, especially with males, and stuff. We work like... I don’t know... we’re not saying that I work faster than females but, you know, it’s just the physical attributes, I guess… - Man RPN 001
**3. Several social identities intersect with gender to shape teamwork interactions**	
Accent/language	I have an example in mind, of fellows that come from distant lands, English is not their first language and I think a natural response that I witness in the OR, even if I try not to practice it myself, is if you talk to them and they don’t seem to understand, the first reaction is to talk louder and then to... there’s almost like… an irresistible urge to treat that person as less intelligent, as less competent. And, and, you know, you always make an effort not to do it, but I do witness it, 'cause we have a number of foreign graduates who work with us. And I think you have to be very, very careful to not let that interfere. – Man anesthesiologist 006
Age	Yeah, I feel like also my age, kind of, because I’m only 24. I’m the youngest nurse so even though I’ve been there two years, people that have started only six months ago that are in their 30’s, even if they’re brand new nurses too, they’re almost more listened to sometimes just because they look older and more mature. – Woman RN 011
Age and experience	I'm a little bit more senior so I think as I become more senior, I've been able to navigate those waters. You start to learn, you know, really that if someone’s upset it's not really about you…You learn these things as you get older. You become less self-centred perhaps. So I think age, I think a certain maturity in your clinical career, a certain comfort with your abilities brings a level of like stand down your anxieties a little bit, you know you can get through this case. –Woman surgeon 003
Age and gender	... you’re often taken less seriously. And I can’t separate whether that’s because I’m young or whether it’s because I’m a female... and I think it’s likely both. I think that young males are taken more seriously than young females are. But I think the older females are also taken more seriously than young females are, so you’re kind of like [expletive]. I think it also... and that’s from everyone, whether it’s kind of conscious or not. – Woman surgeon 008
Experience	...initially on, especially the first few months of residency or medical school or as a junior resident, it’s very difficult to be engaged and feel like you’re part of the team. It’s more like a fly on the wall, kind of how you feel. But with... as the training goes on and in the senior years, the biggest difference I’ve noticed is truly becoming part of the team and my input being valued by various team members and nurses and, and things like that. – Man surgeon 010
Gender and experience	I think junior, you know, when I was sort of being... felt a bit tested, and I don’t know if it was because of a gender thing or if it was because of a junior thing, and I do think that gender had part of it to do with it. It was... these were, you know, older, senior, male surgeons that were doing those sorts of things [challenging decisions]. – Woman anesthesiologist 004
Gender and profession	So, yeah, being a female and also being a nurse, nurses are always kind of underlooked. I think it makes it harder on us to communicate, to be respected, to be heard by everybody. – Woman RN 003
	I was an orderly before, I’ve been with the hospital for 25 years, so but before I became a nurse and stuff. But as a nurse... being a male nurse, I think, compared to being a female nurse, I have an easier time with the dynamics of the surgeons and stuff in the sense that, I have an easier time saying, “Do this. Do that.” And I find there’s still that male/female sort of thing out there in our field, that I get more respect from them and I get less flak from them when I say, you know, “If you want this quicker, do it.” You know, “Stop complaining.”… where I’ve sat there, and I’ve watched my female counterparts sort of do the same thing and they totally brush them off. – Man RN 005
Gender and ethnicity	Well sometimes with a male they can be more dominant…We, let’s say especially Filipinos, we’re more just generally… more of an inferior kind of culture, more of like a humble type low profile, if I may say. So, I guess that also kind of affects how we work in the OR. Not all but just learning to develop a voice in the OR, so sometimes we’re afraid to speak up and speak out, even if we know something is not right. Sometimes we might feel like “Oh this guy won’t listen to me.” – Woman RN 010
Location of training (international medical graduates)	The surgeons might be more welcoming or not as stern with that certain resident [who trained here]… Rather than someone who’s an IMG, like an International Medical Graduate. Doing some sort of, fellowship here… It might be just a subtle practice. People may not even think that they’re doing that. Yeah. Kind of sort of conversations, I’m thinking of conversations or sort of the way they do treat one person over the other. – Woman RPN 009
Ethnicity and language/accent	Yeah. I have witnessed… because we supervise more junior residents and medical students. I would say that there are some cultural barriers as well if there’s a perception that you were not trained within the Canadian system, and often that assumption is made on the basis of race, accent, English as second language sort of perceptions. I would say that there’s more hesitancy towards engaging that person training. – Woman anesthesiologist 002
**4. The influence of gender in the OR can be modified**	
The influence of gender will change with more women in medicine	But things are definitely changing. As there are more and more women in medicine and few... fewer men... I think we don’t have a choice to... to... to... to accept that. To... to... to implement that new reality, basically. – Man anesthesiologist 003
OR culture is improving but there are still inappropriate comments	I think historically I’ve heard more of those kinds of tantrum behaviours and physical outbursts and things that we don’t hear so much of anymore. Of people throwing things and screaming and saying things, yes. That isn’t acceptable. I think to some degree, I mean there might not be those kinds of outrageous acts but I think sometimes there is maybe communications between male doctors and maybe female nurses that still aren’t acceptable. – Woman RN 004
Gender becomes less important once you know each other	And I’d say when people know me more, I think it’s not the case, but I can’t know everyone well and I’d say it’s usually when you’ve… so I’m a subspecialist in thoracic anesthesia so when I work more often with the thoracic surgeon and with the nurses that do more regularly thoracic, I think things work well and there’s no such a thing [as a gender hierarchy]. But sometimes I work in an environment that I don’t go very regularly, and I get… I notice that sometimes… I mean it’s not the same. It’s not like we’re with friends. – Man anesthesiologist 008
Recognition of (white) male privilege	Again, possibly, as a 32-year-old white dude, I’m not... I don’t suffer from, like, a lot of these... not a lot of stereotypes or anything that is affected by my ethnicity or gender or anything like that... it’s something I’ve literally never once in my entire life been like ‘oh, I’ve just been treated that way because I’m a 30-year-old white man’ other than probably good things, other than probably unfair good things, where I’m like, oh, nobody questioned me or bothered me and because... so, it’s something that I’ve noticed, because it’s almost probably universally positive. Unfair, but positive responses that I get directed towards me. – Man surgeon 013
Men can stand up to help women in the OR	So, because the... there’s quite a few females there, like, I was one of maybe five guys in the O.R there. I think me coming in and me sort of standing up for myself about things that they would’ve never done... I think that sort of allowed them to be more comfortable with trying it on their own as well. I think... I think if anything, they’re... you know, trying to stick up for themselves a little bit more... it’s hard to be the only one going against the whole wave of doctors and surgeons, right? But... so I think having more people and sort of teaming up, it... I think it helps. – Man RN 002
Managing unconscious bias	I think that even though there are a lot of people who say that they’re not biased one way or the other sex, I think there’s always an element of potential unconscious bias there. And I think we always need to be aware of and try to manage. – Man anesthesiologist 001

#### Theme 1: Women face challenges in the OR – and everyone experiences or observes it

Both women and men participants observed that women regularly faced challenges in the OR, and they explicitly related these challenges to gender. Across professions, participants indicated that women experienced harassment and bullying, were viewed as less credible, and were not listened to by their colleagues. They also noted that women had to “work so much harder to prove [themselves]” (*Woman RN 14*), feel less respected, and often accept differential treatment as part of their reality.

Women in both anesthesiology and surgery said they were routinely perceived as nurses rather than physicians, which could again undermine their authority. They also reported having to adapt specific strategies in the OR to make their voices heard (e.g. using language carefully, standing on a step stool).

Women in surgery specifically expressed challenges with regard to others’ perceptions of their behaviour. As one woman surgeon explained, “We say that, you know, as a female surgeon, you’re either… a pushover or you’re a bitch…” (*Woman Surgeon 8*). Men surgeons also perceived that assertive behaviour by woman surgeons is “very much likely to be viewed as hostile” while the same behaviour by a man surgeon “would be more likely viewed as a strong leader” (*Man Surgeon 14*). Participants also reported that they have observed the cases of women surgeons being pushed back in order to accommodate a man surgeon’s case first, even when there is no legitimate medical reason (e.g. urgency of the case) (*Man RN 3*).

Tensions between women surgeons and women nurses were identified by participants in each of these groups, and observations of these tensions were also reported by both men nurses and men physicians. Women nurses recognized the challenge of being a woman surgeon in a men-dominated environment, but still felt that this was not a reason for woman surgeons to be “rude” (*Woman RN 6*) to them. Women surgeons reported having to make a conscious effort to befriend woman nurses by acting less confident and less competent because “any sort of confidence and expertise is perceived as a threat” (*Woman Surgeon 7*). The “pushback” from the nursing staff toward women surgeons was observed by participants across professional and gender groups.

Participants also discussed the fact that women are often outnumbered in the OR, particularly in certain specialties such as cardiac surgery. Many participants, both men and women, referred to the OR environment as a “boys club” and considered differential interactions between team members to be a “gender thing”, as one man RN described (*Man RN 5*).

#### Theme 2: Gender impacts team interactions or behaviours

Participants often described the nature of their interactions with other team members as different depending on whether they were working alongside women or men in the OR. For many men nurses and physicians, working with other men was like working with a “buddy” (*Man RN 1*) and communication with each other was “more direct” (*Man RN 2*) than when working with women. Some men nurses commented that being of the same gender as the surgeon can change the typical physician-nurse dynamic. As one man nurse explained,

“…I’m a male who isn’t a doctor in that environment. And it’s… kind of bizarre. ‘Cause it helps, but then it still puts me in kind of a weird position because a lot of the time they’re talking kind of man-to-man with other physicians, and so, when I enter that realm, it’s like I’m one of them but not one of them.” (*Man RN 4*)

Several men participants also reported that they perceived a different “flow” (*Man Surgeon 8*) with regard to the surgical procedure when working with men compared to women. Other men commented that they found it “easier to interact with women [because] they are less likely to try and assume control or be domineering” (*Man Anesthesiologist 5*). Some women described themselves as more “soft spoken” or “less loud” (*Woman Anesthesiologist 2*) than their men colleagues, which affected communication and teamwork for them (e.g. more difficult getting team members’ attention). Conversely, some men recognized that they could “easily be perceived as intimidating” because they are men, and because they do not have “a soft voice” (*Man Anesthesiologist 3*).

#### Theme 3: Several social identities intersect with gender to shape teamwork interactions

Many participants identified additional social identity factors beyond gender that they felt influenced interactions with team members in the OR (e.g. accent or language, age, level of experience, profession, race, ethnicity, and location of medical training).

For many participants, gender could not be separated from other social identities (e.g. age, ethnicity). As one woman surgical post-graduate trainee and one woman nuse commented,

... you’re often taken less seriously. And I can’t separate whether that’s because I’m young or whether it’s because I’m a female... and I think it’s likely both. I think that young males are taken more seriously than young females are. But I think the older females are also taken more seriously than young females are, so you’re kind of like [expletive; screwed] .... and that’s from everyone, whether it’s kind of conscious or not (*Woman Surgeon 4*).

“Being Asian, you know, being an Asian girl, and I look young. Definitely, I’m not treated the same as other people… I just… so I know what I’m walking into, and I’m not going to get upset about it, because it’s just a fact now that’s what I’m going to get most of the time…” (*Woman RN 1*).

Both men and women nurses commented on the interaction between gender and profession in their experience:

So, yeah, being a female and also being a nurse, nurses are always kind of overlooked. I think it makes it harder on us to communicate, to be respected, to be heard by everybody. – *Woman RN 12*

... being a male nurse, I think, compared to being a female nurse, I have an easier time with the dynamic… like, with the dynamics of the surgeons… I get, I believe, more respect from them and I get less flak from them when I say, you know, “If you want this quicker, do it.” You know, “Stop complaining.”…where I’ve sat there, and I’ve watched my female counterparts sort of do the same thing and they totally brush them off. – *Man RN 2*

#### Theme 4: The influence of gender in the OR can be modified

Several participants commented that the influence of gender in the OR was changing, particularly with more women entering medicine. Others felt that while gender often plays a role when individuals first meet, it becomes less important as team members get to know one another. Many men participants, and in particular, men post-graduate trainees, explicitly recognized the privilege they experienced as Caucasian men, and felt that it was important to manage unconscious biases related to gender. As one man anesthesiologist described: “I think there’s always an element of potential unconscious bias there. And I think we always need to be aware of and try to manage.” Men nurses also described their role in helping their women colleagues in the OR. For example, one man nurse commented that “it’s hard to be the only one going against the whole wave of doctors and surgeons”, and that he would help his women colleagues to speak up.

## Discussion

This study examined interprofessional healthcare providers’ perceptions of how gender and additional social identity factors intersect to shape teamwork in the OR. Our results suggest that these factors play a key role in shaping interactions within and between professional groups in the OR. Traditional gender roles, norms and stereotypes [40] are also perceived and experienced by both women and men, but with different consequences. The differential experiences of women and men in the OR observed here may have critical implications for teamwork as they may undermine team morale, communication, and psychological safety, creating an environment conducive to medical errors and adverse events [41–45].

Women and men across each professional group perceived that men were afforded more respect than their women colleagues, regardless of their professional role in the conventional OR hierarchy. Previous studies suggest that gender ability stereotypes exist within surgery, and that surgeons tend to associate men physicians with surgery and women physicians with family medicine [16, 46]. Our results suggest that gender stereotypes extend to other members of the OR team (e.g. anesthesiologists and nurses) and supports the notion that gender is frequently used to categorize others, perhaps even over professional role [47]. For example, men nurses in our study reported a degree of camaraderie with men physicians and felt more respected and listened to by them compared to women nurses *and* women physicians. Conversely, despite belonging to professions typically viewed as leadership roles in the OR, women surgeons and anesthesiologists were routinely challenged by others with regard to their decision-making and leadership qualities. This builds on findings from a previous, smaller study by Pattni and colleagues which found that anesthesia assistants were more likely to challenge the erroneous decisions of a woman anesthesiologist compared to a man anesthesiologist, suggesting that speaking up is influenced by gender within and across the core OR professions [48].

Women anesthesiologists and surgeons in this study often faced challenges in terms of how they were perceived by others, as assertive behaviours were interpreted negatively and resulted in tensions with colleagues. In response to this, many women surgeons in this study reported choosing to minimize their knowledge and skills to appear less threatening. In turn, this could also contribute to them being less respected as surgeons. Conversely, women anesthesiologists reported engaging in particular strategies (e.g. language, positioning) to appear more “in control” and still reported feeling unheard and less respected than their men colleagues. In each case, women physicians experienced difficulty establishing themselves as leaders in the OR, and regardless of the strategy used, did not obtain the desired result. This type of dilemma has been well-documented in other studies, where women in leadership positions have to find a balance between challenging traditional gender roles or conforming to them, with each choice having potential social and career consequences [16, 40, 47, 49, 50]. Future work may further explore the role of gender within the professional socialization of surgeons and anesthesiologists, and how this translates to leadership behaviours. Other research has found evidence of tension and conflict between these disciplines as well as differing norms around and perceptions of teamwork quality, roles and responsibilities in the OR [28, 51–56].

The divergent experiences of women and men in the OR, particularly among physicians, may be one of the contributing factors to the under-representation of women in academic leadership positions, despite beginning residency with similar career goals as their men counterparts.^25^ Gender norms in the OR may be an important contributing social factor to the “confidence crisis” observed among surgical residents [57], and anesthesia residents in this study, which again may affect career trajectories. When women are routinely challenged or not listened to, or perceived negatively for being assertive, as was the case for participants in this study, it may inhibit them from pursuing leadership positions or certain specialties [58]. A recent study of factors influencing career selection for medical students found that occupational segregation of women and men physicians begins as early as the first year of medical school, as both men and women medical students perceive surgery as unwelcoming to women [11]. Further, as implicit gender bias, gendered stereotypes and deeply entrenched structural barriers continue to persist in the surgical field, advancement for women physicians remains a considerable challenge [11].

While gender dynamics in the OR may change as more women enter medicine [59], they are still very much present and particularly influential in specialties that have remained men-dominated, such as orthopedics or cardiac surgery [11, 60]. This could have implications for women’s psychological and occupational (e.g. job satisfaction, burnout) well-being in the OR in addition to teamwork and patient safety [19, 61]. These implications were observed in a recent study in which women surgeons reported interprofessional conflict and breakdowns in communication as a result of gender stereotypes and gendered power relations in the OR, which in turn led to adverse personal, professional, and patient outcomes [18]. Indeed, evidence suggests that breakdowns in teamwork and communication contribute to unintended events such as retained surgical instruments, wrong-site or wrong-procedure, and inadavertenet disease transmission as well as surgical errors resulting in patient injuries and complications [43, 62–65].

Stubborn gender stereotypes and gendered power relations also complicate the hierarchical relationships between professions [66], again posing a threat to effective teamwork. It is noteworthy that tensions were most frequently reported between women nurses and women surgeons. This highlights the pervasiveness and internalization of gender norms, as behaviours exhibited by women and men are often perceived very differently (e.g. “bitchy” vs. “effective leader”) [67, 68] and stereotypes are applied, by both women and men across all social identity categories, to women surgeons [16, 69]. In fact, Braun et al. found that nurses preferred surgeons who displayed stereotypical feminine behaviour (e.g. supportive, nurturing) as opposed to masculine behaviour (e.g., assertive, independent) [70]. Women surgeons may simultaneously feel pressure to maintain professional hierarchies (to establish leadership credibility) and to flatten professional hierarchies (to survive). Often, women physicians in this study discussed needing to “befriend” women nurses to make their lives easier. At the same time, women nurses did not appear to have any issues challenging women surgeons, but expressed fear regarding speaking up against men surgeons. This aligns with findings from previous studies, which reported that women nurses were more willing to “serve” men physicians [71] and that women physicians received less help, support and respect from women nurses compared to their men peers [72]. Interestingly, the tensions observed between women nurses and surgeons were not reported with regard to nurse and anesthesiologist interactions. Future research may further explore the role of gender in physician-nurse relationships in order to better understand why gender appears to be less influential in the anesthesiologist-nurse dynamic compared to the surgeon-nurse dynamic. There may be important lessons to be learned from the former that could be used to develop interventions to improve the latter.

Beyond gender, many additional social identity factors were also revealed to be important in how teamwork was perceived and experienced. It may therefore be important to take an intersectional perspective [73] when designing teamwork interventions in order to address the unique experiences of all OR team members. An intersectional perspective has increasingly been used by researchers to understand the numerous and complex drivers of power relations with regard to patients’ experiences in the healthcare system [74, 75]. With increasing calls for greater attention to diversity and inclusion in surgery [59, 76], it is time to apply this perspective to healthcare professionals themselves, especially in light of the findings reported here. Important factors to simultaneously consider alongside gender include age, accent or language, ethnicity, profession, trainee status, level of experience and location of training. Trainee status, in particular, adds an additional element of complexity with regard to gender and power hierarchies in the OR, and this was certainly emphasized by our participants (e.g., when staff listened to a man resident but not a woman resident, when they each relayed the same information). Other studies have found that residents frequently experience challenges related to hierarchy and speaking up in the OR [5, 6, 77], and it is very likely that resident and staff gender factor into these observations. This is an important direction for future research to consider.

Although the impact of sex and gender on teamwork is complex, it can indeed still be modified. Certainly, interventions to improve elements of teamwork such as communication, psychological safety, trust, and cooperation are unlikely to be optimally effective when negative gender stereotypes and gendered power relations continue to exist in the OR [78]. Thus, a necessary part of improving OR teamwork may first be addressing the role of gender. There are several potential interventions that could be implemented to address the issues raised by participants in our study. For example, simulation sessions could be designed to teach OR team members to recognize and confront implicit biases using specific examples from this study (e.g. the negative perception of women’s leadership behaviours, dismissing the concerns of women who speakup in the OR). The use of in-situ multidisciplinary simulation with natural rather than contrived teams may help to improve the sociological fidelity [79] of these scenarios. Similar principles could also be applied to team debriefings in clinical practice. Teaching OR teams these skills may also help to improve team communication, mutual trust and respect, situation awareness, and psychological safety. Finally, medical school and residency may represent key opportunities to change unequal power dynamics related to gender and the overall professional socialization of healthcare providers. For example, learners may be encouraged to explore the “connection between their personal situations and the structured power relations between privileged and oppressed groups in our society” [80]. This may promote a more informed understanding of the many social factors influencing power relations in the OR, and empower healthcare professionals to challenge and transform gender norms and roles as they enter into practice. Of course, structural reform is also necessary to advance gender equity in medicine, and requires action at the national, provincial and local levels. Flexible scheduling, non-gendered parental leave, child care support, and commitment to principles of equity, diversity, and inclusion in all organizational policies are just some of the many needed changes [81].

### Strengths and limitations

Our study has provided key insights regarding how gender shapes OR teamwork, which can be used to inform future work. Of course, it is important to recognize that there is a spectrum of gender identities, and the experiences of individuals who identify as nonbinary may not be represented here. Our interview question asked participants to self-identify but every participant chose “woman” or “man”. It will be important for future work to recruit and interview gender diverse healthcare professionals regarding their experiences of teamwork in the OR. It should also be noted that the majority of participants in this study were Caucasian. Future work may also employ specific strategies to recruit healthcare professionals who identify as racialized; lesbian, gay, bisexual, two-spirited, queer; differently abled, and so forth, albeit, this can challenging when facing ethics board requirements of maintaining participants’ anonymity.

Although we made specific attempts to recruit men and women from each profession, some groups were still under-represented in this study. There is a need to explore whether the experiences reported by the men nurses in this study are consistent across the broader men nurse population, given the small number of participants. Some surgical specialties, such as general surgery, were over-represented, and this may have been the result of snowball sampling. Future research should determine whether the themes identified by this study are transferable across specialties, as well as across other countries given differences in health systems and potential cultural differences in gender norms and roles. As this was a secondary analysis of a larger study, it will be important to further explore the themes identified here as well as potential new themes in future studies. Nevertheless, a strength of this study is in its large, interprofessional sample size where each of the core OR professional groups (nursing, anesthesia, surgery) was represented.

### Conclusions

Our findings suggest that the highly gendered reality of the OR creates an environment conducive to breakdowns in communication and patient safety risks in addition to diminishing team morale, psychological safety, and provider well-being. Consequently, until teamwork interventions adequately account for gender, they are unlikely to be optimally effective or sustainable.

## Data Availability

The datasets analyzed during the current study are not publicaly available due to participant confidentiality but are available from the corresponding author on reasonable request.

## Abbreviations

COREQ: Consolidated Criteria for Reporting Qualitative Research
OR: Operating room
RN: Registered Nurse
RPN: Registered Practical Nurse
TDF: Theoretical Domains Framework
